# Use of Electroconvulsive Therapy for Treatment‐Resistant Schizophrenia in a Patient Unable to Tolerate Clozapine Due to Clozapine‐Associated Hyponatremia: A Case Report

**DOI:** 10.1155/crps/6269931

**Published:** 2026-08-20

**Authors:** Andrew Hartley, Michael P. Greenage, Justin B. White, Tracey W. Criss

**Affiliations:** ^1^ Virginia Tech Carilion School of Medicine, Roanoke, Virginia, USA, vt.edu; ^2^ Department of Psychiatry and Behavioral Medicine, Carilion Clinic, Roanoke, Virginia, USA, carilionclinic.org

**Keywords:** case report, clozapine, electroconvulsive therapy, hyponatremia, schizophrenia

## Abstract

Clozapine is regarded as the most effective treatment for patients with treatment‐resistant schizophrenia (TRS). However, serious adverse effects such as hyponatremia may necessitate discontinuation, leaving limited therapeutic alternatives. This case report describes a middle‐aged male with chronic schizophrenia who developed clinically significant hyponatremia during clozapine titration, leading to its discontinuation prior to achievement of therapeutic serum concentrations. Despite prior antipsychotic trials with haloperidol, asenapine, olanzapine, and chlorpromazine, the patient had persistent auditory hallucinations and paranoid delusions. Given the patient’s clozapine intolerance and persistent psychosis, a course of bifrontal electroconvulsive therapy (ECT) was initiated three times weekly over a 24‐day inpatient series. The patient demonstrated progressive reduction in auditory hallucinations and paranoid ideation throughout treatment, with resolution of hallucinations and marked improvement in paranoia by discharge. Treatment was well tolerated, with only mild transient headaches and no clinically significant cognitive adverse effects observed on serial mental status examinations. At 5 months post‐ECT, he remained free from readmission and without recurrence of his positive symptoms. This case highlights the potential role of ECT as a therapeutic consideration for select patients with refractory psychosis when clozapine cannot be safely continued due to medically significant adverse effects such as hyponatremia.

## 1. Introduction

Schizophrenia is a chronic psychiatric illness characterized by delusions, hallucinations, disorganized speech, and cognitive impairment. Despite the availability of numerous antipsychotic medications, ~30% of individuals with schizophrenia meet criteria for treatment‐resistant schizophrenia (TRS), defined as inadequate response to at least two different nonclozapine antipsychotic trials of adequate dose, duration, and adherence [1]. These patients are considered to have TRS, which is associated with significant morbidity, poor quality of life, and high societal costs [1]. The mechanisms underlying antipsychotic resistance in schizophrenia are complex and remain an area of ongoing investigation [2]. In a systematic review and meta‐analysis of first‐ and second‐generation antipsychotics, clozapine was superior for treatment‐refractory schizophrenia [3]. However, clozapine’s adverse effect profile, including myocarditis, seizures, metabolic disturbances, and most notably agranulocytosis, can limit its use in certain clinical scenarios.

Hyponatremia is a rare but serious side effect associated with several psychotropic medications. Anticonvulsants and SSRIs are particularly well‐documented causes of hyponatremia, but the link between second‐generation antipsychotics and hyponatremia has not been established with as much conviction [4]. Hyponatremia is a significant concern in clinical practice as it can lead to confusion, seizures, coma, and even death. Thus, monitoring for this complication warrants serious consideration.

In cases of TRS where clozapine must be discontinued, alternative strategies for managing persistent symptoms are required. Electroconvulsive therapy (ECT) has shown potential benefit as an intervention for TRS, particularly in patients who cannot tolerate clozapine or who experience inadequate response to other pharmacologic options [5]. This case report describes a patient with TRS who demonstrated symptomatic improvement with the use of clozapine but developed hyponatremia that necessitated discontinuation of the drug and consideration of alternative treatment avenues. With limited remaining pharmacologic options, ECT emerged as one of the few reasonable therapeutic options available to this patient.

## 2. Case Presentation

The patient is a middle‐aged male with a long‐standing history of psychiatric diagnoses, including bipolar disorder and schizoaffective disorder, who was admitted to inpatient psychiatry for auditory hallucinations and paranoid delusions. During this admission, his diagnosis was revised to schizophrenia, paranoid type. Longitudinal chart review and serial inpatient assessments demonstrated persistent psychotic symptoms occurring outside of discrete mood episodes. These symptoms included chronic auditory hallucinations, persecutory delusions, ideas of reference, and paranoia. The predominance of chronic psychotic symptoms in the absence of sustained manic or depressive episodes during this admission led the treatment team to favor a diagnosis of schizophrenia.

His treatment history prior to admission included chart‐confirmed exposure to haloperidol decanoate, asenapine 2.5 mg, and olanzapine 10 mg. Asenapine was trialed for approximately 2 months but was limited by tolerability and remained below the typical target range. Olanzapine was trialed for ~17 months. Outpatient documentation reflected adherence over this period with repeated symptom recurrence. Although these medications produced partial improvement in anxiety, sleep disturbance, and paranoia, the patient continued to report the persistence of auditory hallucinations, persecutory ideation, and intermittent functional deterioration requiring psychiatric hospitalization. This patient also reported prior trials of aripiprazole and paliperidone, but these medications were unable to be confirmed through a chart review.

On admission, haloperidol was briefly reinitiated due to its availability and efficacy in targeting positive psychotic symptoms. The dosage was started at 2 mg TID PRN. However, this medication was discontinued after several days due to extrapyramidal side effects and poor efficacy in this patient.

The patient was subsequently switched to olanzapine, with titration to 20 mg daily. He was also tapered off carbamazepine (previously prescribed for trigeminal neuralgia) due to concern for olanzapine‐carbamazepine interactions via CYP3A4 induction and transitioned to oxcarbazepine 600 mg BID and pregabalin 75 mg BID. The patient remained severely paranoid and functionally impaired throughout the process of titration. He was also started on sertraline 150 mg daily for comorbid anxiety and obsessive–compulsive features. He was maintained on this regimen for approximately 2 weeks, with no improvement in paranoia or auditory hallucinations. The patient’s baseline predominantly involved daily inquiries about the FBI warrants out for his arrest, as well as auditory hallucinations.

Given this lack of improvement, the treatment team initiated a different antipsychotic. Thorazine (chlorpromazine) 25 mg TID was selected due to its sedating properties and utility in controlling agitation and severe psychotic symptoms. Chlorpromazine was titrated up to 150 mg TID over the course of approximately 3 weeks. While it provided some benefit in reducing agitation, it also was not sufficient for a meaningful reduction of the patient’s paranoia or auditory hallucinations. These symptoms continued to persist and caused substantial distress.

In addition to pharmacologic interventions, throughout this process, the patient was noted to consistently participate in recreational therapy, group therapy, coping‐skills interventions, and milieu‐based treatment. Documentation from therapists and social workers noted appropriate engagement with treatment despite persistent psychotic symptoms.

Given the persistence and severity of psychotic symptoms despite multiple antipsychotic trials and consistent participation in therapeutic interventions, a trial of clozapine was initiated. Clozapine was selected for its well‐established role in TRS, particularly in cases characterized by refractory paranoia and persistent psychotic symptoms. Titration was performed cautiously, beginning at 25 mg QHS with plans to double the dosage every 3 days until a regimen of 200 mg QHS was reached. The patient was also tapered off chlorpromazine simultaneously. During titration, the patient demonstrated progressive improvement in psychotic symptoms, including reduced auditory hallucinations, increased social engagement with staff and peers, and improved participation in unit activities. However, serum clozapine monitoring obtained at a dosage of 100 mg QHS demonstrated a clozapine concentration of 78 ng/mL with a norclozapine concentration of 35 ng/mL. This level is markedly below the commonly cited therapeutic threshold of ~350 ng/mL. Due to concern for rapid CYP1A2‐mediated metabolism, low‐dose fluvoxamine was initiated at 12.5 mg daily to augment clozapine serum concentrations through CYP1A2 inhibition.

**Figure 1:**
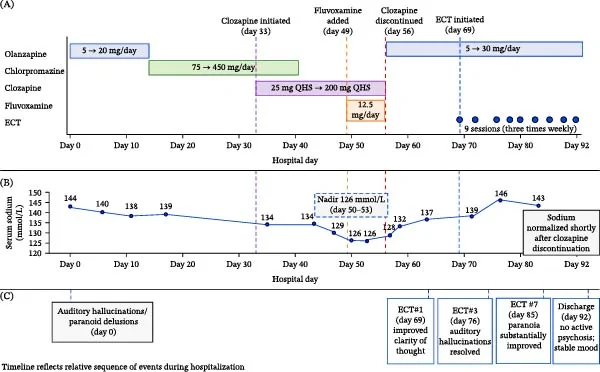
Timeline of psychopharmacologic management, serum sodium trends, and electroconvulsive therapy (ECT) course. (A) Medication timeline. (B) Serum sodium trend. (C) Clinical course/milestones.

Additionally, the patient developed progressively worsening euvolemic hyponatremia during clozapine titration. Serum sodium gradually declined from a baseline of 144 mmol/L to a nadir of 126 mmol/L (Figure 1).

Initial discussions suggested that the hyponatremia was likely multifactorial, with several potential contributing agents being identified. Medications that were considered potential culprits included oxcarbazepine, fluvoxamine, and clozapine [4]. Notably, sodium had already begun to decline before the addition of fluvoxamine, suggesting that clozapine and oxcarbazepine were the more likely primary contributors, with fluvoxamine potentially exacerbating his condition. Despite efforts to mitigate the hyponatremia through salt tablet supplementation and discontinuation of the patient’s hydrochlorothiazide, the electrolyte disturbance persisted. After discussion with the pharmacy, due to the severity and risk associated with progressing hyponatremia, the decision was made to gradually taper and discontinue the patient’s clozapine before repeat serum testing could confirm that therapeutic clozapine concentrations had been reached.

Following the discontinuation of clozapine and reinstatement of olanzapine, the patient experienced a rapid return of baseline psychotic symptoms. This included the resurgence of debilitating paranoia, interpersonal withdrawal, and reduced engagement with staff and other members of the unit. Serum sodium levels normalized shortly after clozapine cessation, strengthening the suspicion that clozapine was contributing to the patient’s hyponatremia. Given his history of treatment‐resistant psychosis and inability to safely continue clozapine, ECT was discussed as another treatment avenue. After detailed discussion of risks, benefits, and alternative treatment options, including explicit review of potential cognitive adverse effects such as transient confusion, anterograde and retrograde memory disturbance, and the possibility of persistent memory effects, the patient provided written informed consent. The capacity for consent was assessed by the treating psychiatrist and judged to be intact. The patient was subsequently tapered off oxcarbazepine and pregabalin to facilitate seizure induction.

Additionally, prior to the initiation of ECT, a second opinion from a board‐certified psychiatrist was completed. The potential benefits of improvement, as well as the risks, were again discussed with the patient. Prior to each treatment, informed consent was conducted by the treating psychiatrist, with the potential benefit of ECT discussed as well as the potential risks, including but not limited to confusion, memory loss, and death.

Treatment was performed using a Thymatron device with general anesthesia. The patient underwent a series of nine bifrontal treatments administered over a 24‐day inpatient course (Figure 1). Initial ECT parameters included pulse width 0.25 ms, frequency 60 Hz, stimulus duration 7.5 s, current 0.90 A, and charge delivery ~201 mC. An ultrabrief pulse width was selected in an effort to reduce cognitive adverse effects while maintaining therapeutic efficacy. Bifrontal electrode placement was selected because the patient was left‐handed, creating uncertainty regarding suitability for right‐unilateral placement due to unknown hemispheric dominance. Thus, bilateral lead placement was chosen secondary to the assessment of the potential benefit of therapeutic efficacy versus the potential risk of cognitive side effects. Typically, the half‐age method of stimulus dosing is utilized to determine the initial stimulus dose [6]. Because concurrent baclofen was expected to elevate the seizure threshold, a slightly higher stimulus dose of 40% was used for the first treatment. This stimulus dose produced adequate generalized seizures throughout the treatment course. Clinical seizure durations ranged from 36–61 s, while EEG seizure durations ranged from 58–130 s.

Throughout the ECT course, the patient demonstrated progressive symptomatic improvement, which was assessed through serial mental status examinations, direct patient reports, and observation documented by the treatment team. While not as sensitive as formal neurocognitive assessments, this documentation allowed for the serial assessment of symptom severity, behavioral functioning, and treatment response. After the first treatment, he reported a moderate reduction in auditory hallucinations and improved clarity of thought. Following the third treatment, the auditory hallucinations resolved. By the seventh treatment, the persecutory delusions and paranoia had substantially improved. Prior to ECT, the patient repeatedly expressed concerns regarding the FBI warrants and beliefs that others were monitoring him. These delusional concerns ceased during treatment and did not recur prior to discharge.

ECT was well tolerated overall. The patient experienced mild transient post‐treatment headaches without prolonged postictal confusion or clinically significant cognitive impairment on serial mental status examinations. Cognitive examinations included clinical assessments of orientation, attention, memory, and language. At discharge, the patient remained on olanzapine 15 mg BID along with sertraline 150 mg daily. Maintenance ECT (m‐ECT) was not initiated at discharge, although this remained a future consideration depending on long‐term clinical stability. At a 5‐month follow‐up, conducted by telephone, the patient endorsed satisfaction with his current mental state and denied recurrence of paranoia or auditory hallucinations. He reported continued adherence to outpatient pharmacotherapy, no emergency department visits, no psychiatric rehospitalizations, and stable housing.

## 3. Discussion

This case highlights the potential association between clozapine titration and clinically significant hyponatremia in the setting of complex psychiatric polypharmacy. It also emphasizes the prospective utility of ECT for select patients with treatment‐resistant psychosis who cannot safely continue clozapine. Clozapine remains the most effective agent for refractory psychosis, with literature demonstrating superior outcomes when compared to other antipsychotic medications [3]. Still, alternative treatments must be accessible for patients who cannot tolerate the side effects of clozapine. In this scenario, the failure of clozapine and other first‐line antipsychotics in the management of schizophrenia led to a difficult clinical impasse.

ECT has historically been viewed as a treatment of last resort for schizophrenia. However, emerging literature suggests that ECT may be underutilized and could be beneficial in certain subtypes of schizophrenia, including those with catatonia, prominent negative symptoms, or clozapine resistance. In patients who cannot tolerate clozapine, ECT may partially recapitulate its therapeutic benefits. A study by Petrides et al. [5] found that ECT was associated with response rates over 50% in clozapine‐resistant schizophrenia, and this benefit also extended to those unable to tolerate the drug.

ECT is generally considered safe when performed under modern guidelines. Potential cognitive side effects include transient confusion and disturbances in anterograde and retrograde memory. Many patients experience improvement in these symptoms over time, although persistent memory complaints have also been reported and should be discussed during informed consent. The patient described in this case report tolerated treatment well, experienced symptomatic improvement, and demonstrated no clinically apparent cognitive impairment on serial mental status examination, though the absence of formal neurocognitive testing should be noted.

Several limitations should be acknowledged. Moderate quality evidence indicates ECT has a positive effect for TRS, but more good‐quality evidence is needed [7]. A recent randomized, double‐blind, sham‐controlled trial of ECT in clozapine‐resistant schizophrenia by Melzer‐Ribeiro et al. [8] did not demonstrate superiority of active over sham ECT, underscoring the continuing uncertainty about the general efficacy in this population. Case reports such as this one can add descriptive observations to the literature but cannot remedy the methodological gaps that randomized, controlled trials are needed to address.

Beyond questions of acute efficacy, sustaining response after an acute ECT course also remains a substantial challenge in TRS. A recent systematic review and meta‐analysis by Aoki et al. [9] reported pooled relapse proportions following acute‐course ECT in schizophrenia of ~37% at 6 months, 41% at 12 months, and 55% at 24 months. The addition of continuation or m‐ECT to antipsychotic therapy was associated with substantially lower 6‐month relapse rates (~20%), supporting its role in sustaining response after acute treatment. In this case, m‐ECT was discussed but not initiated at discharge given the patient’s robust acute response, personal preferences, geographical constraints, and transportation barriers.

Last, this report describes a single patient experience and therefore cannot establish causality between clozapine titration and hyponatremia or determine the generalizable efficacy of ECT in TRS. Concurrently, formal psychometric instruments such as the Positive and Negative Syndrome Scale (PANSS) or Brief Psychiatric Rating Scale (BPRS) were not serially administered during hospitalization, limiting objective quantification of symptom severity and treatment response. Formal neuropsychological testing was also not performed, and a comprehensive assessment of psychosocial contributors to the patient’s presentation was not undertaken during hospitalization. Clinical improvement and cognitive tolerability were assessed primarily through serial psychiatric examinations, behavioral observations documented throughout hospitalization, and through direct patient communication after discharge.

## 4. Conclusion

ECT may represent a reasonable therapeutic consideration for carefully selected patients with TRS who cannot tolerate clozapine due to its medically significant adverse effects. Although additional research is needed to better define its role in schizophrenia, this case demonstrates the potential for ECT to provide meaningful symptomatic improvement when conventional pharmacologic strategies are limited by intolerance or inadequate response. This present case report was prepared in accordance with the CARE guidelines (Supporting Information).

## Author Contributions

Andrew Hartley drafted the manuscript and conducted the literature review. Tracey W. Criss and Justin B. White provided critical revisions and supervised the manuscript development. Michael P. Greenage reviewed the manuscript for clinical accuracy and contributed to patient care.

## Funding

No specific funding was received for this case report.

## Disclosure

All authors have approved the final manuscript. All clinical content, interpretation, and manuscript authorship remain the work of the listed authors, who reviewed and approved the final text and take full responsibility for the accuracy and integrity of the manuscript.

## Ethics Statement

This case report was prepared in accordance with the ethical principles of the Declaration of Helsinki (1964, as revised in 2013) and follows the International Committee of Medical Journal Editors (ICMJE) recommendations for case report publication. Formal Institutional Review Board (IRB) approval was not required for this retrospective single‐patient case report per institutional policy at Carilion Clinic. All identifying information has been removed from the case description in accordance with the ICMJE guidelines to protect patient confidentiality.

## Consent

Written informed consent for the publication of this case report was obtained from the patient. The signed consent form is securely archived by the corresponding author and is available to the journal or relevant ethics committee upon request.

## Conflicts of Interest

The authors declare no conflicts of interest.

## Supporting Information

Additional supporting information can be found online in the Supporting Information section.

## Supporting information


**Supporting Information** The case report was prepared in accordance with the CARE guidelines. A completed CARE checklist has been provided as Supporting Information.

## Data Availability

The data supporting the findings of this case are available within the article. Further deidentified data are available from the corresponding author upon reasonable request and are subject to institutional data‐sharing policies and patient confidentiality protections.
