# Supplementary material for: Use of Electroconvulsive Therapy for Treatment‐Resistant Schizophrenia in a Patient Unable to Tolerate Clozapine Due to Clozapine‐Associated Hyponatremia: A Case Report
Source: Case Rep Psychiatry. 2026 Aug 20;2026:6269931. doi: 10.1155/crps/6269931 (PMC13492052; doi:10.1155/crps/6269931)
Supplement: Supplementary file 1 [file CRPS-2026-6269931-s001.pdf]

CARE case report guidelines

CARE Checklist of information to include when writing a case report

(cc) BY-NC-ND

Topic	Item	Checklist item description	Reported on Line
Title	1	The diagnosis or intervention of primary focus followed by the words "case report"	1-2
Key Words	2	2 to 5 key words that identify diagnoses or interventions in this case report, including "case report"	40
Abstract (no references)	3a	Introduction: What is unique about this case and what does it add to the scientific literature?	34-38
	3b	Main symptoms and/or important clinical findings	27-28
	3c	The main diagnoses, therapeutic interventions, and outcomes	28-33
	3d	Conclusion—What is the main "take-away" lesson(s) from this case?	36-38
Introduction	4	One or two paragraphs summarizing why this case is unique (**may include references**)	56-70
Patient Information	5a	De-identified patient specific information.	73-75
	5b	Primary concerns and symptoms of the patient.	78-81
	5c	Medical, family, and psycho-social history including relevant genetic information	83-92
	5d	Relevant past interventions with outcomes	83-92
Clinical Findings	6	Describe significant physical examination (PE) and important clinical findings.	73-75
Timeline	7	Historical and current information from this episode of care organized as a timeline	Figure 1
Diagnostic Assessment	8a	Diagnostic testing (such as PE, laboratory testing, imaging, surveys).	127-138, 185-187
	8b	Diagnostic challenges (such as access to testing, financial, or cultural)	87-90
	8c	Diagnosis (including other diagnoses considered)	73-81
	8d	Prognosis (such as staging in oncology) where applicable	N/A
Therapeutic Intervention	9a	Types of therapeutic intervention (such as pharmacologic, surgical, preventive, self-care)	94-183
	9b	Administration of therapeutic intervention (such as dosage, strength, duration)	94-183,199-203
	9c	Changes in therapeutic intervention (with rationale)	94-183
Follow-up and Outcomes	10a	Clinician and patient-assessed outcomes (if available)	201-205
	10b	Important follow-up diagnostic and other test results	N/A
	10c	Intervention adherence and tolerability (How was this assessed?)	196-205
	10d	Adverse and unanticipated events	196-198
Discussion	11a	A scientific discussion of the strengths AND limitations associated with this case report	209-261
	11b	Discussion of the relevant medical literature **with references**.	209-261
	11c	The scientific rationale for any conclusions (including assessment of possible causes)	265-269
	11d	The primary "take-away" lessons of this case report (without references) in a one paragraph conclusion	265-269
Patient Perspective	12	The patient should share their perspective in one to two paragraphs on the treatment(s) they received	N/A
Informed Consent	13	Did the patient give informed consent? Please provide if requested	Yes ☒ No ☐
